# Single Lipid Molecule Dynamics on Supported Lipid Bilayers with Membrane Curvature

**DOI:** 10.3390/membranes7010015

**Published:** 2017-03-15

**Authors:** Philip P. Cheney, Alan W. Weisgerber, Alec M. Feuerbach, Michelle K. Knowles

**Affiliations:** 1 Department of Pharmaceutical Sciences, University of Colorado Anschutz Medical Campus, Aurora, CO 80045, USA; philip.cheney@ucdenver.edu; 2 Department of Chemistry and Biochemistry, University of Denver, Denver, CO 80208, USA; Alan.Weisgerber@du.edu (A.W.W.); feuerbacham@gmail.com (A.M.F.)

**Keywords:** supported lipid bilayers, membrane curvature, lipid sorting, single molecule tracking, colocalization, quantitative imaging, lipid

## Abstract

The plasma membrane is a highly compartmentalized, dynamic material and this organization is essential for a wide variety of cellular processes. Nanoscale domains allow proteins to organize for cell signaling, endo- and exocytosis, and other essential processes. Even in the absence of proteins, lipids have the ability to organize into domains as a result of a variety of chemical and physical interactions. One feature of membranes that affects lipid domain formation is membrane curvature. To directly test the role of curvature in lipid sorting, we measured the accumulation of two similar lipids, 1,2-Dihexadecanoyl-*sn*-glycero-3-phosphoethanolamine (DHPE) and hexadecanoic acid (HDA), using a supported lipid bilayer that was assembled over a nanopatterned surface to obtain regions of membrane curvature. Both lipids studied contain 16 carbon, saturated tails and a head group tag for fluorescence microscopy measurements. The accumulation of lipids at curvatures ranging from 28 nm to 55 nm radii was measured and fluorescein labeled DHPE accumulated more than fluorescein labeled HDA at regions of membrane curvature. We then tested whether single biotinylated DHPE molecules sense curvature using single particle tracking methods. Similar to groups of fluorescein labeled DHPE accumulating at curvature, the dynamics of single molecules of biotinylated DHPE was also affected by membrane curvature and highly confined motion was observed.

## 1. Introduction

Membranes are organized into nanoscale domains of lipids and proteins for optimal physiological function [1]. These domains act to accumulate the protein machinery needed for a variety of essential cellular processes, such as secretion [2,3,4] and signaling [5,6], and the local lipid environment directly affects the function of a variety of ion channels [7,8]. Like membrane and membrane-associated proteins, lipids also sort within the cell. On the cell surface, domains enriched with sphingolipids and cholesterol are thought to form ordered domains [1]. Whereas, within the cell, different membrane compartments contain different lipid compositions, with the trans-Golgi network actively sorting sphingolipids and sterols for delivery to the plasma membrane [9]. Interestingly, exogenously added lipids also sort and the tails of the lipids are a key feature by which they can be organized [10]. Overall, cells are highly heterogeneous in their distribution of proteins and lipids.

To better grasp the mechanisms by which membrane associated molecules are organized in cells, supported lipid bilayers have been used for approximately 30 years as a simple mimic for cellular membranes [11]. Supported lipid bilayers are chemically tunable, fluid, and amenable to fluorescence imaging methods. Even in bilayers containing only simple lipid mixtures, in the absence of proteins, lipid sorting is observed [12,13,14,15]. A thorough and recent review describes the two mechanisms that can drive lipid sorting in lipid-only systems [16]. These include lipid–lipid phase separation, where lipids organize based on similar tail saturation and the sorted domains are stabilized by cholesterol. In this mechanism, a multi-component mixture of lipids is required and changes in membrane shape can both spontaneously occur and stabilize domains [17]. The second mechanism by which lipid microdomains form requires lipid mixtures that are not globally phase-separated, but rather have micro-emulsions or regions where lipids locally separate. These micro-emulsions can be stabilized in a variety of ways, including the addition of surfactants that ease the line tension at the interface between lipid domains or by local regions of membrane curvature [16]. In both mechanisms, membrane shape plays a role in sorting lipids or stabilizing lipid domains.

Membrane shape has recently been identified as an instigator and stabilizer of lipid domain formation [18,19,20,21,22,23,24], but the mechanism by which lipids sort into curved regions is not clear. Curvature based lipid sorting has been observed in experimental work where curved tubular membranes are created from giant unilamellar vesicles [22,25] and in curved bilayers [26] for lipid mixtures. In these experiments, curvature aids in phase separation. One model for curvature assisted lipid sorting suggests that lipids are recruited based on their intrinsic molecular shape. However, the coupling between molecular shape and membrane shape is likely very weak [27]. Another way to sort lipids at regions of curvature is by the flexibility of domains. A disordered lipid domain has higher flexibility than an ordered domain and takes less energy to bend. Finally, a third model depends on the formation of defect sites in a lipid bilayer [23,28]. Hydrophobic curvature packing defects form as a flat bilayer is bent and these defect sites are potential binding locations for defect sensing molecules. In each of these mechanisms it is likely that the addition of protein stabilizes lipid domains formed on regions of membrane curvature [22].

Although accumulation of proteins and lipids at curvature is observed in a variety of experiments [23,29,30,31,32,33], single molecules have demonstrably different behavior [33]. So far, single proteins have not been observed accumulating at curvature, suggesting that a cluster of molecules is needed for accumulation or that the affinity for curvature is quite low and hard to observe in single molecule experiments, where the concentration is inherently low. This is demonstrated in research on the membrane associated protein amphiphysin [34] and the epsin N-terminal homology (ENTH) domain [33]. The curvature association of these proteins depends on the amount bound to the membrane with a higher concentration leading to more accumulation of protein at curvature. However, a variety of membrane associated proteins have been studied, and no experiments address the interactions between single lipid molecules and membrane curvature.

In this work, we measure the dynamics of lipids at regions of membrane curvature and flat regions on a supported lipid bilayer where the curved and flat regions are connected. As opposed to single liposome based methods for measuring proteins at curvature [23], molecules within a bilayer can diffuse from one region of curvature to another. The motivation for designing this curved, lipid surface is to better mimic the cell surface, which is thought to be ruffled. With a biochemical mimic, the shape and chemical composition are separately controlled and lipid accumulation can be directly attributed to the shape in the membrane.

**Figure 1:**
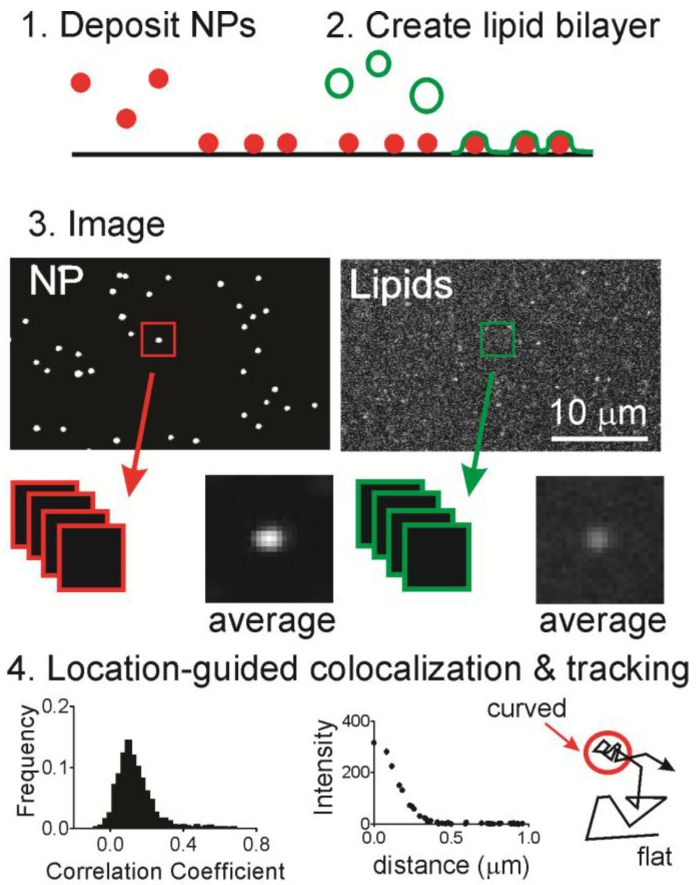
The general scheme of the experiment and analysis. (Step **1**) Fluorescent NPs are deposited on a clean glass surface followed by (Step **2**) incubation with liposomes for the preparation of a supported lipid bilayer; (Step **3**) fluorescence microscopy is performed and images of NPs and fluorescently labeled lipids are sequentially obtained at the same location. NP positions are located and both images are cropped at these positions to give pairs of images; and (Step **4**) the Pearson’s correlation coefficient is calculated for each pair of images, the average plot of intensity as a distance from the center pixel (termed a “radial plot”), and single particle tracking is performed on the two-color data (described left to right).

Here, we use a nanoparticle-patterned substrate that supports a lipid bilayer to create spatially isolated and readily identified regions of membrane curvature (Figure 1). The membrane shape can be adjusted by changing the nanoparticle substrate and the chemical composition of the bilayer is separately tuned. Using fluorescence microscopy methods, we measured the mobility and localization of a common two-tailed lipid, DHPE, with either a biotin-streptavidin or fluorescein molecule attached to the head group. A fluorescein labeled single tailed fatty acid of the same length (16 carbon saturated tail) was also measured (hexadecanoic acid or HDA). Accumulation of lipids at sites of curvature was observed for both fluorescein labeled DHPE (Fl-DHPE) and HDA (Fl-HDA), with Fl-DHPE accumulating more. Single molecule experiments also show that the dynamics of a molecule depend on whether or not it is located at a region of curvature. Molecules at curvature move, but are highly confined. Molecules on flat regions are very mobile but avoid moving onto regions of curvature. Overall, lipids accumulate at sites of curvature, but single molecules do not accumulate more at curvature or easily transition from curved to flat regions.

## 2. Results

To determine whether lipids accumulate at curvature, we used a supported lipid bilayer that contained localized regions of membrane curvature (Figure 1). Fluorescent nanoparticles (NPs) were deposited onto a cleaned glass surface (Figure 1, Step 1) and then liposomes containing fluorescently labeled or biotinylated lipids were incubated in the solution above at 37 °C for one hour (Figure 1, Step 2), during which time the liposomes fused with the surface to form an extended bilayer [35]. One key feature of this system is the lack of lipid–lipid phase separation. The bilayer contains 99% palmitoyl-2-oleoyl-*sn*-glycero-3-phosphocholine (POPC) and 1% Fl-DHPE or Fl-HDA. Note that all lipid chemical structures are shown in Figure S1. The only unique features capable of causing lipid sorting are the curved regions. After incubation and the removal of any unfused liposomes, fluorescence microscopy was performed to determine if labeled lipids co-localize with regions of curvature (Figure 1, Step 3).

To determine if labeled lipids accumulated at curved regions, the two-color images were cropped around every nanoparticle position and then analyzed in two ways (Figure 1, Step 4). First, the Pearson’s correlation coefficient was calculated to determine the extent of similarity in pairs of cropped images. A perfect match would have a coefficient of 1.0 and the inverse of an image would have a coefficient of −1.0. A score of 0 means there is no correlation. The second, quantitative measurement calculated from the cropped lipid images is the radial plot. This is a measurement of the intensity as a function of the distance from the center, where the region of curvature is located. From a series of images of single molecule dynamics, DHPE molecules were tracked relative to NP positions in time.

### 2.1. Bilayer Characterization

**Figure 2:**
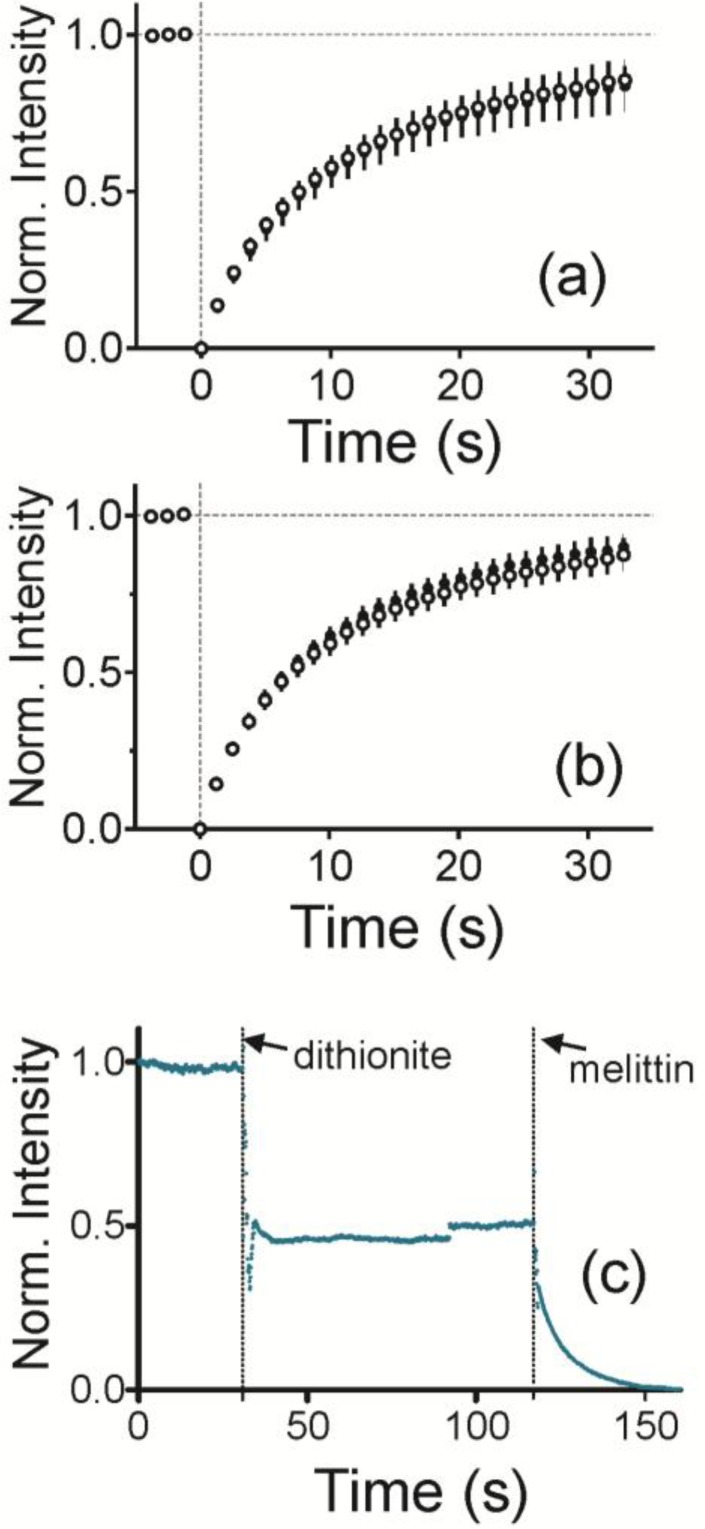
FRAP recovery for a 10 µm diameter region photobleached at time 0 s. (**a**) Fl-HDA (white circles) and Fl-DHPE (black circles) on samples that contain 28 nm ROC; (**b**) Fl-HDA (white circles) and Fl-DHPE (black circles) on samples that contain 55 nm ROC. Error bars are standard deviation; (**c**) NBD-DHPE fluorescence is quenched by dithionite. Once melittin, a pore forming protein, is added, fluorescence decreases to background levels.

To characterize the curved, supported lipid bilayer, lipid fluidity and the presence of a bilayer were measured. To measure fluidity, fluorescence recovery after photobleaching (FRAP) traces was measured for each sample. The average and standard deviation are plotted in Figure 2a for a radius of curvature (ROC) of 28 nm and in Figure 2b for an ROC of 55 nm. Bilayers are fluid when NPs are present and Fl-HDA and Fl-DHPE recover equally well.

To obtain quantitative information about the dynamics of lipids, the fluorescence recovery traces were fitted according to previously established protocols as shown in Equations (1) and (2) [36]. Fluorescence recovery data, Y (τ), was fit with a single exponential with the following equation:

Y (τ) =Y_0_ + (Y_infinity_ − Y_0_)(1 − exp(−kτ)) (1)

where Y is the intensity within the bleached region at times, τ, 0, and ∞. To obtain diffusion coefficients, the time to recover to half the intensity is calculated according to: τ_1/2_ = ln (k/2). From this, the diffusion coefficient is:

D = (*r*^2^/4τ_1/2_)γ_D_ (2)

where γ_D_ = 0.88 for a circular bleached area and r is the radius of the bleached spot. The fitting results are summarized in Table 1. Overall, there are no significant differences in any of the curves. This is partly due to having relatively few NPs in the bleach area; the NP density in these experiments is <0.01 NP/µm^2^. Most of the dynamics measured in the FRAP assay relate to the fluidity of the lipids on the flat, glass surface.

To determine if the bilayer is two layers and impermeable, NBD labeled lipids were incorporated into both sides of the bilayer and then quenched with sodium dithionite (Figure 2c). As dithionite is not able to pass through an intact lipid bilayer, this reaction is used to demonstrate access to a leaflet of the bilayer [37]. This is observed by a drop in fluorescence upon addition of dithionite at 30 s. Fluorescence levels were off by up to approximately 48% of the initial fluorescence. Once melittin, a pore forming protein, is added, dithionite gains access to the leaflet closest to the solid support. This reduces the fluorescence to the background level.

### 2.2. Lipid Accumulation at Membrane Curvature

**Figure 3:**
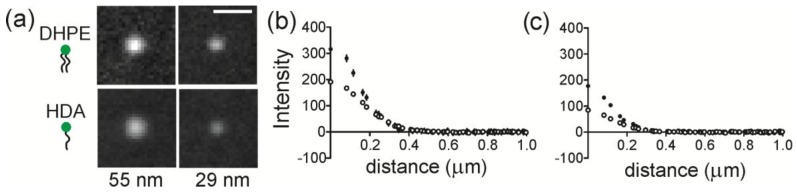
Location-guided colocalization measurements of fluorescent lipids at regions of membrane curvature. (**a**) cropped and averaged images for each size and lipid measured. All images have background subtracted and are scaled identically. Scale bar = 1.0 µm; (**b**) a radial plot of Fl-DHPE (black circles) and Fl-HDA (white circles) at regions with 55 nm radius of curvature; (**c**) a radial plot of Fl-DHPE (black circles) and Fl-HDA (white circles) at regions with 28 nm radius of curvature. The error bars represent the standard error of the mean (SEM).

The accumulation of lipids at regions of membrane curvature was measured by quantifying the intensity of lipids at NP regions. The average images of Fl-DHPE and Fl-HDA cropped around NP locations are shown in Figure 3a. Here, the background has been subtracted and all images are scaled linearly and identically for direct, visual comparison. Fl-DHPE collected at a radius of curvature (ROC) of 55 nm in the highest amount and Fl-HDA collected in the least amount at an ROC of 28 nm. The projection of the membrane shape likely accounts for most of the differences between different curvatures, but the shape is consistent from one lipid to another, making the different lipids directly comparable. To quantify the intensity, a radial plot was calculated from each cropped image and the average radial plot is shown for DHPE and HDA at an ROC of 55 nm (Figure 3b) and for an ROC of 28 nm (Figure 3c). For both sizes, Fl-DHPE accumulated more at curvature than Fl-HDA.

**Figure 4:**
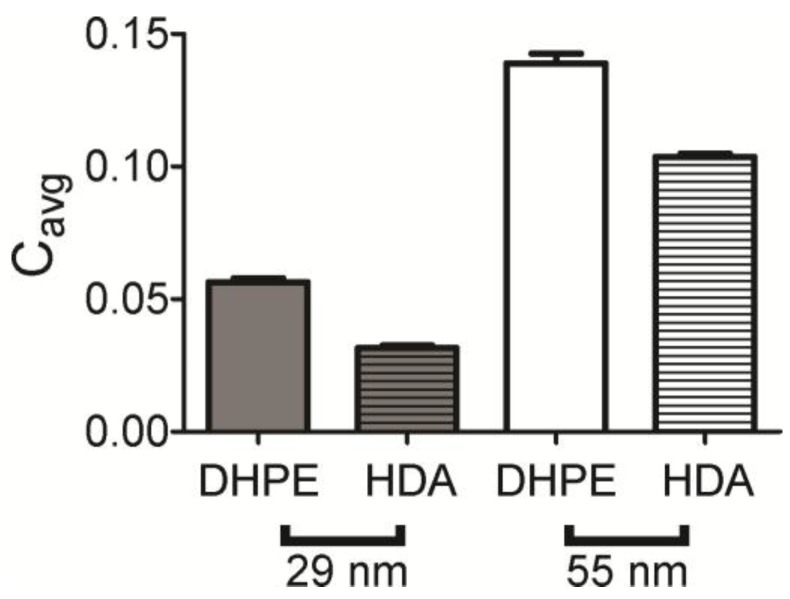
Pearson’s correlation coefficient was calculated for NP: lipid pairs of images that were cropped from the same sample region. DHPE (solid bars) accumulates more than HDA (striped bars).

The radial plot is a direct measure of the amount of labeled lipid present, but a second measurement of colocalization was calculated from pairs of images. Here, the Pearson’s correlation coefficient was measured for each lipid–NP image pair and averaged. The full distribution is shown in Figure S2 and the average is shown in Figure 4. This suggests that lipids accumulate at curvature for all lipids and shapes measured, and the trend is similar to that observed in Figure 3 with DHPE colocalizing the most and HDA the least. Although the Pearson’s correlation coefficients are quite small, DHPE is significantly higher than HDA, indicating that the lipids are weakly associated with curvature, but DHPE associates more. It is interesting to note that in our bilayer system, the bilayer composition is symmetric and higher degrees of sorting have been observed when we added lipids specifically to the upper leaflet only [18].

### 2.3. Single Molecule Lipid Dynamics

**Figure 5:**
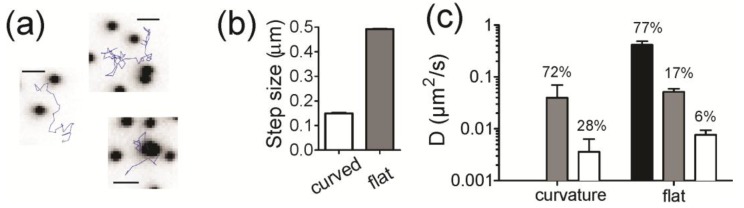
Single Biotin-X-DHPE molecules tagged with Streptavidin-Alexa546 were tracked on the curved supported lipid bilayers (ROC = 28 nm) in space and time. (**a**) example trajectories (blue) show the heterogeneous dynamics observed on both flat (white) and curved (black) regions. Scale bar = 1 µm; (**b**) the average step a molecule takes over 0.228 s (5 frames) when starting at a region of curvature (white) or at a flat region (grey); (**c**) the distribution of steps observed at curved and flat regions (shown in Figure S3) was fitted to Equation 3 to obtain the diffusion coefficients and the percentage of steps moving at that rate. The average D is plotted for *t* = 0.091, 0.228 and 0.456. Note that there is no fast component for the tracks that start at regions of curvature.

To address the dynamics of lipids specifically at regions of membrane curvature, we used single molecule imaging and tracking methods. The highest colocalizing lipid (Fl-DHPE) was chosen and is also commercially available with a biotin linkage in place of the fluorescein. However, since the imaging modality for single molecules is total internal reflection fluorescence (TIRF) microscopy, we only measured lipid bilayers containing small (ROC = 28 nm) features to stay within the excitation range (~100 nm depth) of the TIRF field. To prepare samples for single molecule imaging, Biotin-X-DHPE (0.1%) was incorporated into curved bilayers containing POPC and then tagged in situ with fluorescently labeled streptavidin (Strep-546). Figure 5a shows example trajectories that were observed at flat and curved regions of membrane, where the black dots are the NPs and the blue lines are the tracks of a single lipid. At first glance, molecules at curvature are more confined. To quantify the dynamics of trajectories, we calculated the amplitude of the displacements made for single lipids at regions of curvature and compared that to the displacements of single lipids on flat regions (Figure 5b,c). For a track to be considered to be at curvature, the position of the lipid must be within two pixels (214 nm) of the position of an NP and colocalization with curvature is, therefore, time dependent. Parts of a track can be colocalized, whereas other parts can be on flat regions. The steps of the track that start at curvature are counted as colocalized. Tracks are significantly more confined when at curvature, exhibiting more than a three-fold reduction in step amplitude relative to tracks on flat regions (Figure 5b).

The distribution of step sizes (Figure S3) was fitted with the following equation to determine what fraction of the molecules were moving at certain speeds [38]:

(3) $$Y\;\left(r,t\right)=r\left[A_{1}\;exp\left(-\frac{r^{2}}{4D_{1}t}\right)+A_{2}\;exp\left(-\frac{r^{2}}{4D_{2}t}\right)+\;A_{3}\;exp\left(-\frac{r^{2}}{4D_{3}t}\right)\right]$$

Three modes of motion were used to fit the dynamics of tracks on the flat regions of the surface, similar to what has been done previously [39]. The three modes of motion are: immobile (Figure 5c, white), slow (Figure 5c, grey) and fast (Figure 5c, black) The dynamics of lipid molecules on the regions of curvature, however, fit well with only two parameters (immobile and slow); the high speed motion observed on flat regions was not present in tracks that started at a curved regions. The diffusion coefficients from the fit results are summarized in Figure 5c and the contribution of each rate of diffusion is written as a percentage above the bars. Overall, the motion of lipids at regions of curvature is confined.

## 3. Discussion

One purpose of this work was to examine how different lipids sort as a function of membrane shape. Two lipids were tested; Fl-DHPE has two 16 carbon-long, saturated tails and Fl-HDA has one 16 carbon long, saturated tail. For both radii of membrane curvature measured (28 nm and 55 nm), Fl-HDA accumulates less to curved regions, but all lipids show some accumulation (Figure 3 and Figure 4). Three models were put forth regarding the mechanism of lipid sorting at curvature. The first mechanism relies on the intrinsic shape of molecules. Cylindrically shaped lipid molecules, where the head and the tail of the lipids are approximately equal in occupied volume, prefer flat regions. DHPE and POPC are considered to be cylindrical lipids. Inverse conical shaped molecules, where the lipid head group takes up more volume than the lipid tails, prefer to sort into positively shaped curvature [40]. Single tailed lipids, like lysoPC and Fl-HDA, are inverse conical in shape. Although the coupling between membrane shape and molecular shape is weak [27], we would expect that Fl-HDA would accumulate more at curvature that Fl-DHPE and this was not observed (Figure 3 and Figure 4).

The second mechanism of lipid sorting at curvature depends on phase separation, where the flexible, disordered lipid domains could be bent over the nanoparticles more easily than ordered lipid domains. In our system, lipid phase separation is not likely to occur as POPC makes up over 97% of the lipid composition.

The third mechanism of lipid sorting at regions of curvature involves a defect site model. In this model, defect sites in the membrane form as it curves, creating locations within the lipid bilayer where hydrophobic tails are more exposed to the external buffer [23,28]. The number of defect sites increases with higher curvatures (smaller ROC) and these serve as binding locations for other molecules with exposed hydrophobic portions, such as palmitoylated proteins, lipids, or amphipathic helices [23]. The amount bound to curvature depends on two factors: the number of binding sites and the affinity of the molecule for a defect site. Since the number of binding sites is unlikely to be different when comparing Fl-HDA and Fl-DHPE on the same membrane shape, affinity for defect sites likely plays a role, with Fl-DHPE having higher affinity for defect sites than Fl-HDA. Our results agree with past work comparing Fl-DHPE and Fl-HDA on single liposomes [23], suggesting that the support beneath the bilayer is not playing a role in sorting. In liposome based work, Fl-DHPE accumulated more on smaller liposomes and a trend was observed between the number of carbons in the tails and the amount that accumulates, with longer or multiple lipid tails accumulating more. Finally, the head group could play a larger role in lipid sorting than we have previously acknowledged. The differences between the HDA linkage and DHPE linkage to fluorescein are not identical (structures can be found in Figure S1). However, the role of the head group in sorting was measured previously and showed little effect on curvature sensing [23].

Even though sorting occurs with DHPE lipids, single molecule trajectories do not show preference to accumulate at curvature. Approximately 1.2% of the tracks observed resided within two pixels of an NP location. With an average of 404 NPs, each creating a 214 nm radius circle of area considered to be colocalized within a 3000 µm^2^ area for the movies analyzed, approximately 7.8% of the surface contains regions of colocalization. This suggests that molecules avoid curvature. It is also difficult to find trajectories that transition from flat to curved regions, yet FRAP data has shown that lipids’ NP positions recover [18] and the single molecule tracking of SB-DHPE clearly demonstrates that movement of molecules at sites of curvature is very confined (Figure 5).

The lack of single molecule tracks favoring sites of curvature and colocalizing with nanoparticles is in agreement with the work of others, where ENTH proteins sorted to positive curvature on a wavy supported lipid bilayer, but when single proteins were tracked, no preference was observed [33]. Two alternative reasons why the fraction of single molecule tracks that colocalize to curvature is so low could be due to: (1) the resolution of the microscope. If two molecules were contained within the same region of membrane curvature, they would be counted as one track; (2) particles that did not move during the entire image sequence were excluded from the analysis and this may also lower the percent of lipids that were considered to be colocalized. Future experiments using photo-activatable probes or other methods of analysis could elucidate this further.

Even though the number of tracks that colocalized with curvature was lower than expected, single lipid molecules that resided at curved regions were affected by the membrane shape (Figure 5). The dynamics were confined, but molecules at sites of curvature likely escape to exchange over the course of minutes as we observed previously with FRAP measurements [18]. The rates of diffusion measured here for single lipids are slower than what has been measured previously by others [39]. For example, single molecule tracking experiments of a PIP2 binding protein domain measured a fast diffusion coefficient of 1.4 µm^2^/s on flat, supported bilayers when two lipid molecules were bound. This is approximately 2.5 times faster than our observations for SB-DHPE. One major difference in our experiments is the use of POPC instead of DOPC. POPC has a higher melting transition temperature (−2 °C) compared to DOPC (−17 °C) and is more viscous [41]. A second reason could be due to the fact that streptavidin acts as a cross-linker to lipids and has four biotin binding sites. However, only two are likely available on one side of the protein. If more lipids bind, movement would also be slower.

## 4. Materials and Methods

### 4.1. Curved Supported Lipid Bilayer Materials and Preparation

All fluorescent nanoparticles, fluorescently labeled lipids, biotinylated lipids and fluorescently labeled streptavidin were purchased from Life Technologies, Carlsbad, CA, USA. This includes: 5-hexadecanoyl-aminofluorescein (Fl-HDA), *N*-(Fluorescein-5-thiocarbonyl)-1,2-dihexadecanoyl-*sn*-glycero-3-phosphoethanolamine (Fl-DHPE), *N*-((6-(biotinoyl)amino)hexanoyl)-1,2-dihexadecanoyl-*sn*-glycero-3-phosphoethanolamine (biotin-X-DHPE), Streptavidin-Alexa 547 (Strep-546), Marina Blue 1,2-dihexadecanoyl-*sn*-glycero-3-phosphoethanolamine (MB-DHPE). 1-Palmitoyl-2-oleoyl-*sn*-glycero-3-phosphocholine (POPC) was purchased from Avanti Lipids, Alabaster, AL, USA. Buffer and glass cleaning reagents were purchased from Sigma Aldrich, St. Louis, MO, USA.

Nanoparticle patterned substrates were created by depositing fluorescent fluospheres on a cleaned glass surface followed by liposome deposition [18]. Specifically, 8-well glass dishes (Lab-Tek Chambered Borosilicate Coverglass System, Thermo Fisher, Waltham, MA, USA) were cleaned by washing in 0.1% sodium dodecyl sulfate (SDS), deionized water and then 1% bleach, followed by storage in deionized water. On the day of bilayer preparation, 8-well dishes were cleaned with 2% Hellmanex, Hellma Analytics, Müllheim, Germany, for one hour, followed by extensive rinsing with buffer. The buffer were used for all experiments was 4-(2-hydroxyethyl)-1-piperazineethanesulfonic acid (HEPES) buffer (30 mM HEPES, 140 mM NaCl, 2 mM CaCl_2_, pH 7.4).

After cleaning, yellow-green fluorescent (505 nm excitation/515 nm emission, 45 nm diameter) or red fluorescent (580 nm excitation/605 nm emission, 48 or 100 nm diameter) carboxylate modified polystyrene nanoparticles (Thermo Fisher) were deposited on the glass slide. The nanoparticles were then covered with a lipid bilayer using standard liposome deposition techniques [35]. This involves making liposomes by probe sonication of lipid films (0.125 mM lipids in buffer). Liposomes were extruded through a 100 nm filter (Avanti Polar Lipids, Alabaster, AL, USA) and then incubated with the nanopatterned surface for 1 h at 37 °C. The increased temperature was necessary and fluid bilayers were not obtained at room temperature with our deposition conditions. Membrane fluidity was tested with FRAP, using Fl-DHPE or Fl-HDA, or by imaging single molecule motion using total internal reflection fluorescence microscopy. Confocal microscopy was performed to measure colocalization between nanoparticles and lipids.

In confocal imaging measurements, the lipid bilayers contained 98% POPC and either Fl-DHPE (2%) or Fl-HDA (2%). In single molecule imaging measurements, the lipid bilayers contained POPC (97.9%), MB-DHPE (2%) and biotin-X-DHPE (0.1%). The dynamics of Biotin-X-DHPE lipids were detected by in situ labeling with Alexa Fluor 546 (Thermo Fisher) conjugated to streptavidin (Strep-546). The concentration of labeled lipid used was determined by the amount needed for FRAP experiments on MB-DHPE and then kept constant for all samples.

The term “radius of curvature” (ROC) is calculated by the diameter of the NP plus the thickness of a bilayer (5 nm added to each side) and then divided by 2 to obtain the radius. For 100 nm NPs deposited, this corresponds to a ROC of 55 nm; for 45 nm NPs deposited, this corresponds to 28 nm.

### 4.2. Confocal Microscopy

To measure membrane fluidity and colocalization between NPs and labeled lipids, a point-scanning confocal microscope (Olympus Fluoview, Olympus, Center Valley, PA, USA) capable of fluorescence recovery after photobleaching (FRAP) was used. For two color imaging, the red and green fluorescence were taken sequentially with a 100× objective set at 3× zoom such that one pixel was equal to 82 nm. The red channel containing NPs was excited with a 559 nm laser and emission was collected from 575 to 675 nm. The green channel was excited with the 488 nm line of an Argon ion laser and emission was collected from 500 to 545 nm. Images of nanoparticles and labeled lipids were acquired at a rate of 12.5 µs/pixel. When FRAP was performed, a 488 nm laser was used to excite and photobleach fluorescein labeled lipids in a 10.25 µm diameter circular area. FRAP sequences were acquired with a dwell time of 2 µs/pixel and a size of 512 × 512 pixels. All imaging was performed at approximately 23–25 °C.

FRAP sequences were analyzed using ImageJ (version 1.50, National Institutes of Health, Bethesda, MA, USA) and Prism (version 7.0, GraphPad, La Jolla, CA, USA). All frames were adjusted for photobleaching by comparing to a reference area outside the bleached area. The FRAP average intensities were normalized to the highest (usually pre-bleach images) and lowest values (the bleach image) by dividing by the highest intensity and subtracting the lowest. The diffusion coefficient (D) and the mobile fraction was calculated by fitting, as described in the results [36].

### 4.3. Total Internal Reflection Fluorescence (TIRF) Microscopy

Single particle tracking experiments were performed using a TIRF microscope (Nikon, Tokyo, Japan) equipped with a 60× 1.45 NA objective and a 2.5× magnifier for a final resolution of 107 nm/pixel. A 491 nm laser was used to excite the NPs, and a 561 nm laser was used to excite of the Strep-546. Emission was detected on an EMCCD camera (Andor iXon 897+, Andor Technology Ltd., Belfast, UK). The nanoparticles remain stationary and an image was taken by exciting with 491 nm prior to dynamic measurements of the protein tagged lipids at 561 nm. A dual-color, TIRF dichroic was used (Chroma, Bellows Falls, VT, USA) with emission filters (Omega Optical, Brattleboro, VT, USA) at 525/45 nm and 595/60 nm. The dichroic filter passed both the green and red fluorescence and was specifically engineered for TIRF imaging to maintain beam quality. Image series were captured with an exposure time of 30.28 ms and a frame-to-frame interval of 45.6 ms using µManager [42]. 

### 4.4. Single Molecule Tracking Analysis

Thirty-five movies of lipid bilayers containing labeled SB-DHPE were analyzed in MATLAB (R2016b, MathWorks, Natick, MA, USA) using routines adapted from Blair and Dufresne [43], which are based on commonly used, particle tracking routines [44]. To analyze the movie data, individual images were filtered and locations identified with sub-pixel resolution. Locations were connected from frame to frame in a way that minimizes the total displacement of particles. Particles that did not move during the entire image sequence were excluded from the analysis. Each track location was compared to the NP positions, which were also found with sub-pixel resolution after band-pass filtering, and marked as colocalized if the lipid particle positions were waiting within 2 pixels of the stationary NPs. From the tracks, the amplitude of the displacement was calculated, as described previously [45]. Diffusion coefficients were then calculated from displacement histograms of colocalized and non-colocalized as described in the results.

### 4.5. Location-Guided Colocalization Image Analysis

Location-guided colocalization methods were used and these methods are visually summarized in Figure 1. Two main calculations were performed: a radial plot and Pearson’s correlation function. Both of these analyses begin in the following way: (1) fluorescent NPs, which mark locations of curvature, were identified using spot-finding methods based on freely available code [43]; (2) NP locations were kept if they were further than 9 pixels of another NP or further than 12 pixels from the image edge. The distances were chosen based on the average NP separation and the cropped image size; (3) images were cropped from these location in both the NP and the lipid channel. Afterwards, the distribution of Pearson’s correlation coefficients was calculated for each pair of images, and the average radial plot of the cropped lipid images. This was done by averaging all pixels that are a specific distance from the center pixel. MATLAB code for the radial averaging function is available upon request. The correlation function calculation was obtained from Dr. Justin Taraska [46]. Pearson’s correlation coefficient was calculated according to:

$$c=\frac{\sum_{=1\;}^{n}\left(R_{i}-\langle R\rangle \right)\left(G_{i}-\langle G\rangle \right)}{\sqrt{\sum_{i=1\;}^{n}\left(R_{i}-\langle R\rangle \right)}\sqrt{\sum_{i=1\;}^{n}\left(G_{i}-\langle G\rangle \right)}}$$

where *R* is the red cropped image, *G* is the green cropped image and *R_i_* is the intensity of the *R* image at pixel number *i*. The function sums over all pixels (*n*) in an image.

### 4.6. Melittin-Based Quenching Assay

Supported lipid bilayers were formed as described above with POPC:NBD-DHPE (2-(4-nitro-2,1,3-benzoxadiazol-7-yl)aminoethyl-DHPE (Avanti Polar Lipids, Alabaster, AL, USA)) at a 98:2 molar ratio on a nanoparticle patterned substrate containing red fluorescent 100 nm fluospheres (Thermo Fisher). TIRF images of the NBD fluorescence were measured in time at 10 Hz and sodium dithionite (5 mM in HEPES buffer) was added to quench the NBD dye after 30 s of imaging. The focus slightly drifts upon the addition but was refocused. At 117 s pore forming protein, melittin (Sigma Aldrich, St. Louis, MO, USA) was added for a final concentration of 1.76 µM. The average image intensity was measured for each time point and normalized by dividing by the first image intensity value.

GraphPad Prism and Microsoft Excel (version 2013, Redmond, WA, USA) were used for all other plots and data fitting and significance testing.

## 5. Conclusions

In conclusion, this work demonstrates a simple system for created curved lipid bilayer using a nanoparticle patterned surface. At regions of curvature, two-tailed (DHPE) and one-tailed (HDA) molecules accumulate at curvature, with two tailed molecules accumulating more. When single molecules are tracked, DHPE molecules do not favor sites of curvature. Instead, the curvature acts to contain molecules within the curved region and exclude those on the flat regions, creating a local, nanoscale confinement zone for lipids.

## Figures and Tables

**Table 1:** Diffusion coefficients and fraction mobile measured from FRAP recovery curves.

ROC (nm)	Lipid	D (µm^2^/s)	% Mobile
55	Fl-DHPE	0.89 ± 0.02	84
28	Fl-DHPE	0.93 ± 0.03	90
55	Fl-HDA	0.89 ± 0.03	86
28	Fl-HDA	0.92 ± 0.03	87

## Supplementary Materials

The following are available online at http://www.mdpi.com/2077-0375/7/1/15/s1, Figure S1: Chemical structures of lipids used in the study; Figure S2: Distributions of the Pearson’s correlation coefficient for HDA and DHPE at regions of membrane curvature; Figure S3: Distributions of the displacements made by single Strep-DHPE molecules.
