# Supplementary material for: Single Lipid Molecule Dynamics on Supported Lipid Bilayers with Membrane Curvature
Source: Membranes (Basel). 2017 Mar 15;7(1):15. doi: 10.3390/membranes7010015 (PMC5371976; doi:10.3390/membranes7010015)
Supplement: Supplementary file 1 [file membranes-07-00015-s001.pdf]

**Supplemental Figures**

Single Lipid Molecule Dynamics on Supported Lipid Bilayers with Membrane Curvature by Cheney *et.al.*

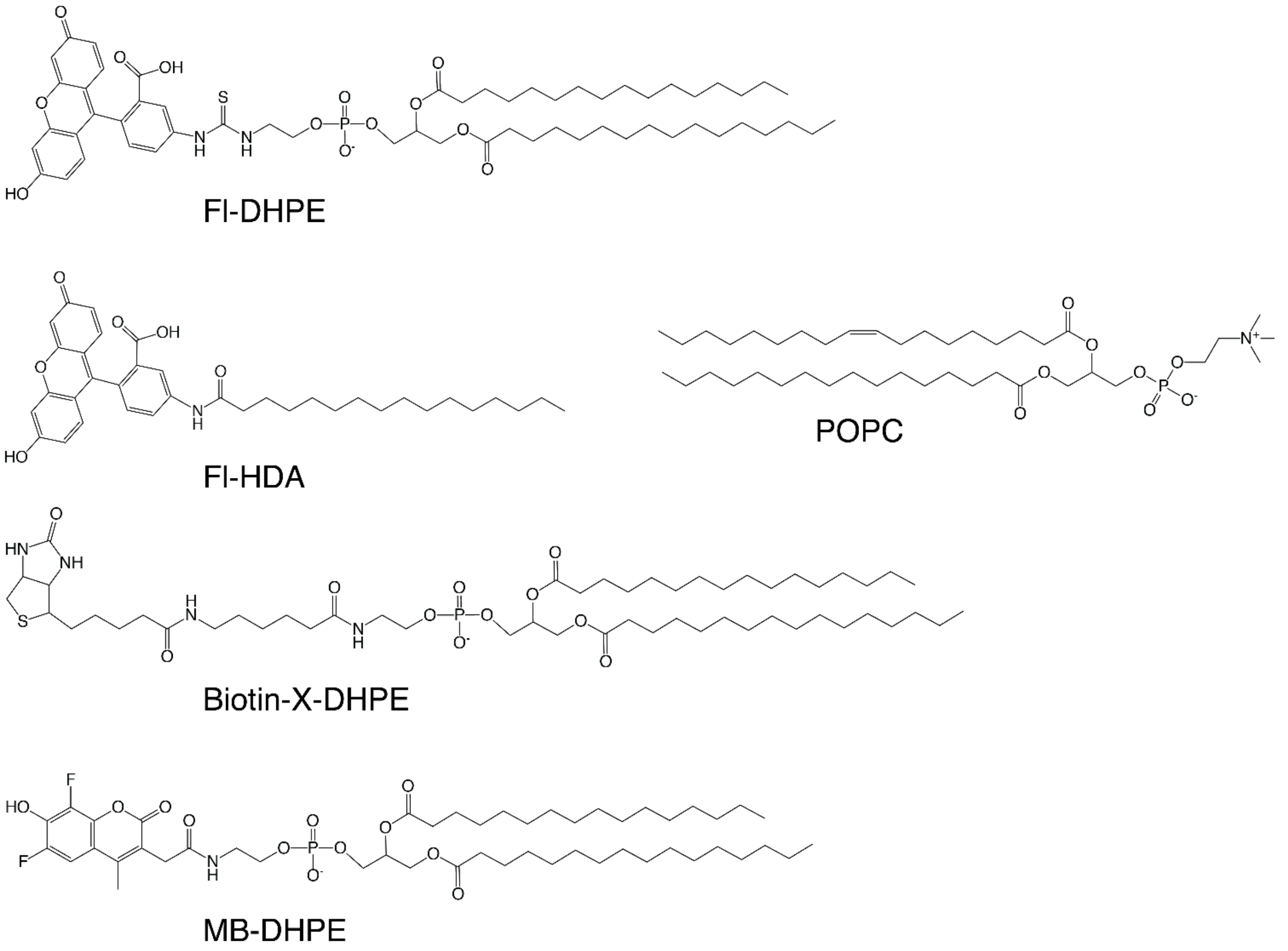


**Figure S1:** Chemical structures of the lipids used in this study. Fluorescein labeled DHPE (Fl-DHPE, Thermo Fisher Cat. Num. F362), Fluorescein labeled Hexadecanoic Acid (Fl-HDA, Thermo Fisher Cat. Num. H110), Biotin labeled DHPE (Biotin-X-DHPE, Thermo Fisher Cat. Num. B1616), Marina Blue® labeled DHPE (MB-DHPE, Thermo Fisher Cat. Num. M12652) and POPC (Avanti Polar Lipids Cat. Num. 850457).

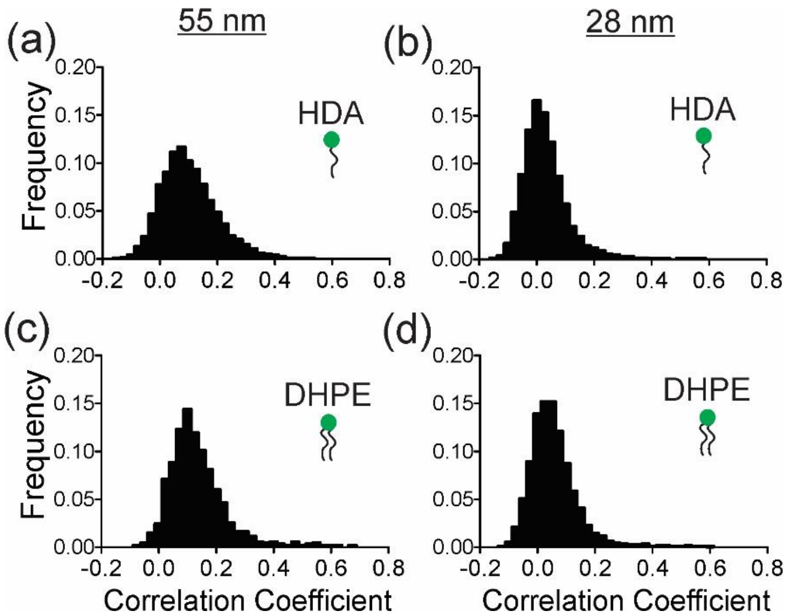


**Figure S2:** Distributions of the Pearson's correlation coefficient for HDA and DHPE at regions of membrane curvature. A) Fl-HDA at regions of curvature that have a ROC pf 55 nm. B) Fl-HDA at regions of curvature that have a ROC pf 28 nm. C) Fl-DHPE at regions of curvature that have a ROC pf 55 nm. D) Fl-DHPE at regions of curvature that have a ROC pf 28 nm.

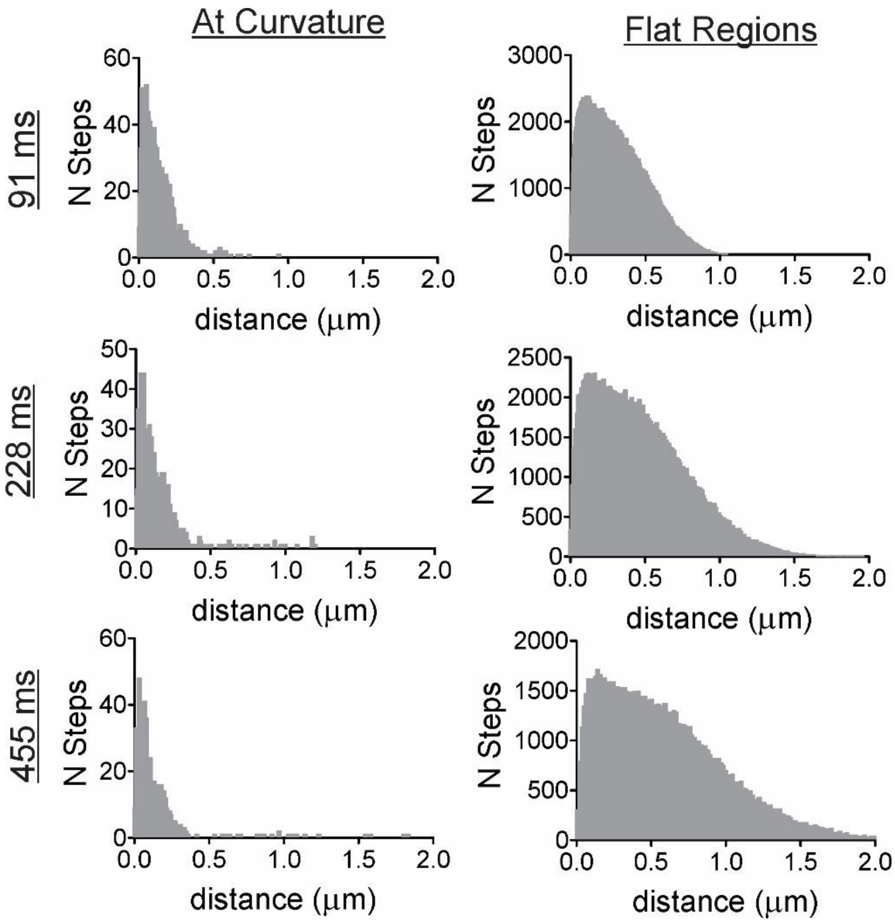


**Figure S3:** Distributions of the displacements made by single Strep-DHPE molecules. Tracks were separated into steps that start at curved regions (left) and flat regions (right) for 91, 228 and 455 ms time delays.
